# The age distribution of cancer and a multi-stage theory of carcinogenesis

**DOI:** 10.1038/sj.bjc.6602297

**Published:** 2004-12-21

**Authors:** P Armitage, R Doll

**Affiliations:** 1 From the Statistical Research Unit of the Medical Research Council, London School of Hygiene, Keppel Street, London W.C.I.

First published in *British Journal of Cancer* (1954) **8**, 1–12

ATTEMPTS to derive theoretical laws from changes in the death rate with age have a long history. They have not, in general, been very fruitful and there has been some hesitation in applying the technique to the study of cancer. Recently, however, two hypotheses about the mechanism of carcinogenesis have been put forward, which have been derived from analysis of cancer mortality statistics. Fisher and Hollomon (1951) used statistics from the United States for cancer of the stomach in women, and Nordling (1953), classing all sites together, used statistics for cancer in men from Britain, France, Norway and the USA. Both found that, within the age group 25–74 years, the logarithm of the death rate increased in direct proportion to the logarithm of the age, but about six times as rapidly; in other words, the death rate increased proportionally with the sixth power of the age. Death rates in some age groups under 25 years were higher than would be expected had this basis been a general law throughout life. Rates for the age groups above 75 years were considered unreliable and were excluded.

In interpreting these observations, both assumed that mortality gave a good indication of incidence and treated the data as if they referred to age specific incidence rates. They considered that cancer in youth might be affected by special factors which were unlikely to operate at later ages and they, therefore, felt justified in basing their hypotheses on the data recorded for adults.

Fisher and Hollomon (1951) pointed out that the observed relationship between age and mortality could result if a colony of six or seven cancer cells was a critical size below which independent growth was not sustained. This hypothesis, however, also leads to the conclusion that cancer incidence should be proportional to the fifth or sixth power of the concentration of the effective carcinogen whereas experimental data suggest that, in general, tumour incidence and concentration of the carcinogen vary in arithmetical proportion. The hypothesis in its simple form is, therefore, untenable.

Nordling (1953), on the other hand, suggested that the observed relationship would be explained if a cancer cell was the end-result of seven successive mutations. This hypothesis does not lead to the observed result in all circumstances. It does so only if the probability of occurrence of each mutation remains constant throughout life. In this case—and so long as the occurrence of each mutation is a relatively rare event—the incidence rate of cancer at age *t* will be proportional to the probabilities of occurrence of each mutation per unit time and the sixth power of the age,


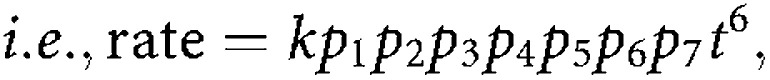


*k* being a constant term (see Mathematical Appendix). The logarithm of the incidence rate will then be directly proportional to the logarithm of the age and will increase six times as rapidly,


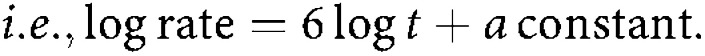


In contrast to Fisher and Hollomon's (1951) hypothesis this leads to the conclusion that the final rate of tumour formation will be directly proportional to the concentration of an applied carcinogen, so long as the probability of occurrence of a mutation is proportional to the concentration of the carcinogenic factor and different factors are required to effect different mutations.

In his analysis, Nordling (1953) grouped all types of cancer in men together and considered them as a whole. He gave no detailed figures for cancer in women, but stated that for cancer of the sex organs the increase in mortality was fairly small above the age of 45 years and that for other sites the mortality seemed “to increase according to the sixth power of the age both before and after the forties but not during the decade of the menopause when the increase is smaller”. He considered that the entire increase with age in cancer incidence among adults was not wholly explicable by a mechanism of multiple mutations but that hormonal control of growth might play an independent part. The purpose of the present paper is to examine the relation between mortality and age for cancer of different sites for each sex and to see whether Nordling 1953 hypothesis can account for the data when it is recognized that the strength of the carcinogenic factors may be variable. It will, however, not be assumed that the successive cellular changes leading to the development of cancer are necessarily mutations (as was postulated by Nordling (1953)), since there is evidence that carcinogenic and mutagenic activity are not always identical (Berenblum and Shubik, 1949) and since the nature of the cellular change is irrelevant to the mathematical analysis. All that need be postulated is that the changes of state should be specific and discrete and that each stage should be stable. It will be assumed further that the changes must proceed in a unique order.

Figures 1

**Figure 1:**
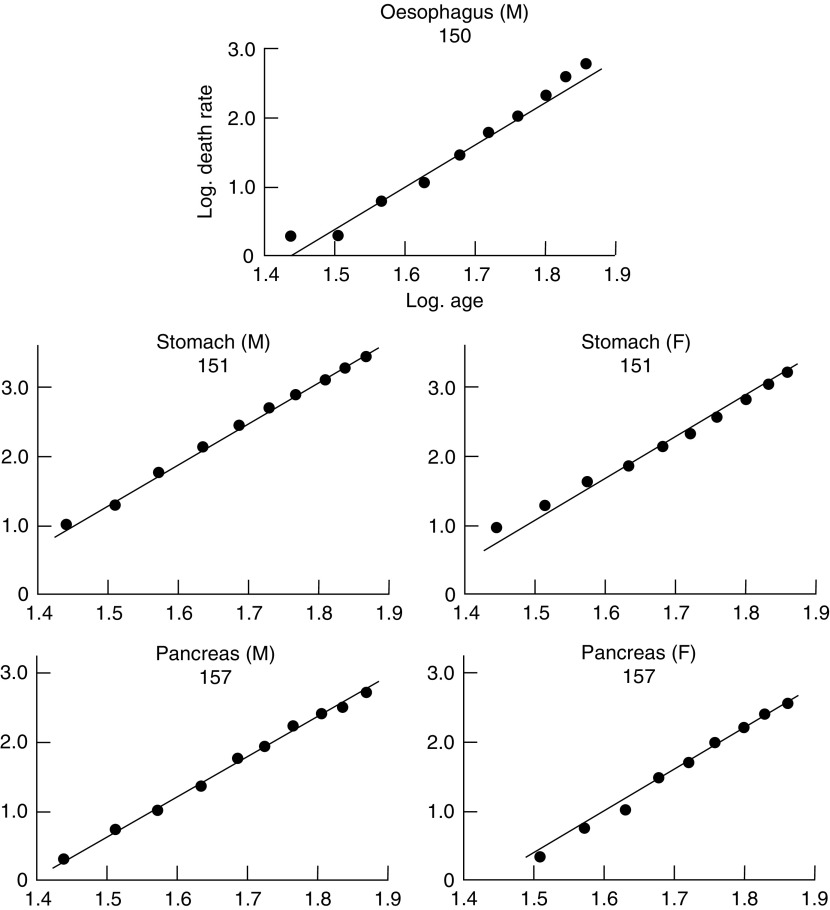
Change in mortality with age for cancer of the oesophagus, stomach and pancreas in men and for cancer of the stomach and pancreas in women shown on a double logarithmic scale, that is, the logarithm of the death rate per million persons plotted against the logarithm of the mid-point of the age group. The straight line through the points has been drawn arbitrarily to give the best fit, subject to the gradient being 6 to 1.

, 2

**Figure 2:**
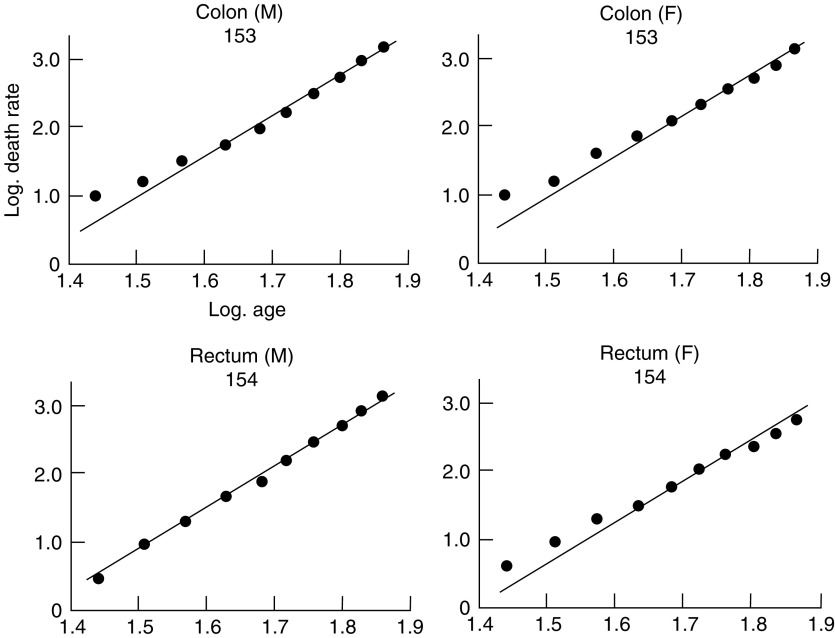
Change in mortality with age for cancer of the colon and rectum in men and women, shown as in Figure 1.

, 3

**Figure 3:**
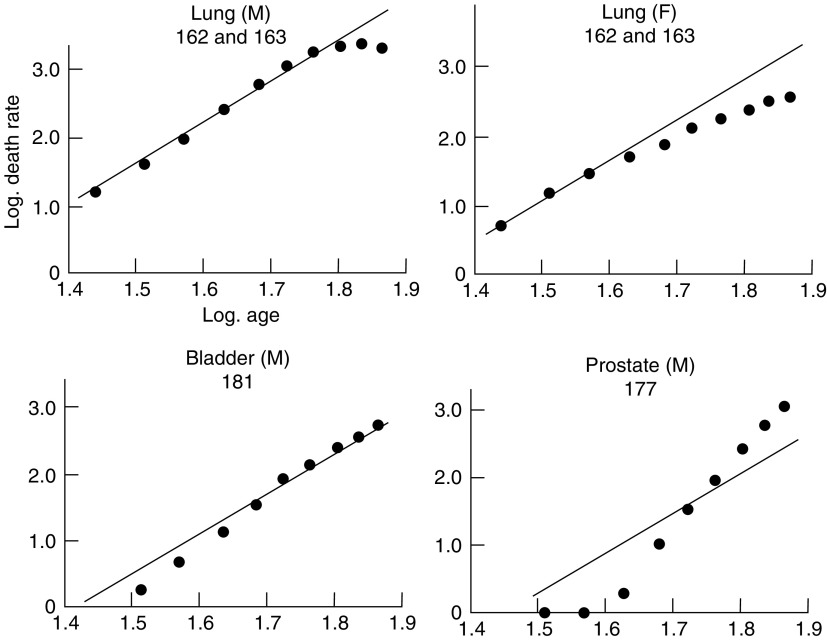
Change in mortality with age for cancer of the lung, bladder and prostate in men and for cancer of the lung in women, shown as in Figure 1.

and 4

**Figure 4:**
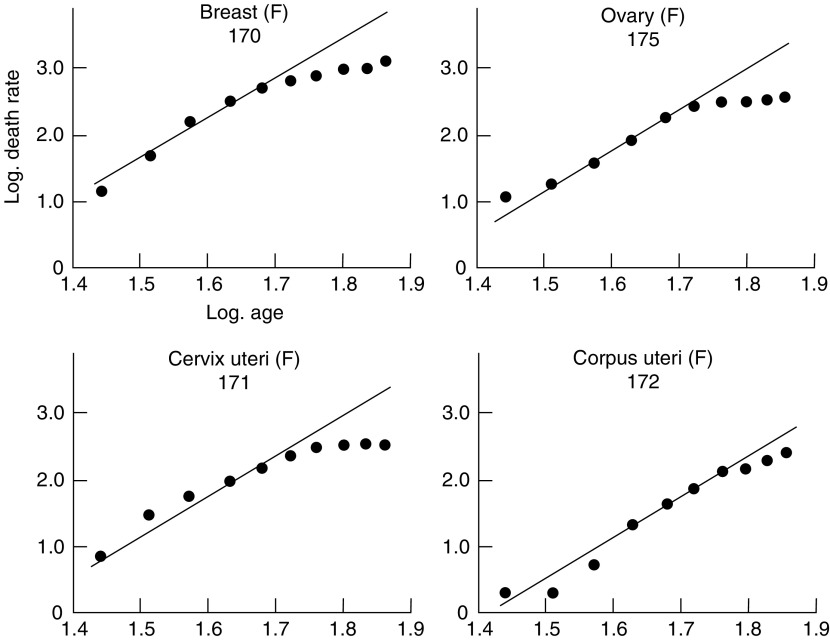
Change in mortality with age for cancer of the breast, ovary and cervix and corpus uteri in women, shown as in Figure 1.

show the logarithms of the male and female death rates from cancer of the commonest sites in England and Wales in 1950 and 1951 (Registrar General, 1952, 1953), plotted against the logarithms of the mid-points of the age groups. For each sex, all those sites are shown for which more than 1000 deaths were recorded in each of the two years. The rates are plotted for five-year age groups between the limits of 25 and 74 years. The rates for the first two or three age groups are less reliable than those for the subsequent ones since they are based on much smaller numbers, and both standard errors and (to a lesser extent) arithmetical errors are much larger. For example, the standard error of the death rate from gastric cancer in women is ±18 per cent of the rate for the first age group (25–29 years) and ±2 per cent of the rate for the last (70–74 years). That is to say, the logarithms of the true rates for these age groups are likely to lie within a range of ±0.13 and ±0.02 respectively of those shown in Fig. 1.

Straight lines with a gradient of 6 to 1 (i.e. 6 units increase in the logarithm of the death rate per unit increase in the logarithm of the age) have been drawn through each set of observations. From inspection of the graphs, it is seen that the different types of cancer fall into two main groups. In the first—composed of cancer of the oesophagus, stomach, colon, rectum and pancreas in men and of cancer of the stomach, colon, rectum and pancreas in women—the observations fall fairly close to the lines. In some, however (cancer of the colon in men, cancer of the stomach, colon and rectum in women), the observations fall closer to lines with less steep gradients. The relatively high rates at the younger ages could, however, result if the population contained a group of subjects specially susceptible to cancer of these sites in early life—and so far as cancer of the colon and rectum is concerned, such a group is in fact found in subjects of polyposis coli.

The actual regression coefficients for all the first group of cancers are shown in Table 1

**Table 1:** Regression of the logarithm of the death rate from cancer of selected sites on the logarithm of the age

**Sex**	**Site of cancer**	**Regression coefficient of logarithm of death rate on logarithm of age**
M	Oesophagus	6.26
	Stomach	5.91
	Colon	5.18
	Rectum	5.62
	Pancreas	5.76
		
F	Stomach	5.27
	Colon	4.97
	Rectum	5.03
	Pancreas	6.48

; they vary from 4.97 to 6.48. All the mortality rates for this group, therefore, show the same sort of association with age as was reported by Fisher and Hollomon (1951) and by Nordling (1953). The data can be said to accord with the theory that 6 or 7 successive changes in the cell are necessary before cancer appears as a clinical entity, so long as the probability of occurrence of each change is assumed to remain constant throughout life. It is notable that the cancers concerned are, in fact, those which are likely to be independent of hormonal control and—with the exception of cancer of the oesophagus—those for which no changing environmental factors have been suspected.

In the second group of cancers—composed of cancer of the lung, bladder and prostate in men and cancer of the lung, breast, ovary and cervix and corpus uteri in women—the observations plotted in Fig. 3 and 4 diverge to a great extent from the hypothetical regression lines, wholly or in part. This group, however, consists of cancers for which there is already reason to believe that the strengths of some of the factors responsible for their development are variable. Cancers of the prostate, breast, ovary and cervix and corpus uteri are all believed to be influenced by endocrine secretions, which vary in each individual in the course of his life; a proportion of the cases of cancer of the lung is believed to be related to cigarette smoking, which has become more prevalent in the last 50 years and a proportion of the cases of cancer of the bladder is due to occupational hazards, to which men have been exposed for various periods at various ages. On the hypothesis that carcinogenesis is a multistage process, therefore, cancer at these sites would not be expected to show a uniform relationship between death rates and any power of the age. On the contrary, departures from the hypothetical lines such as are shown in Fig. 3 and 4 would necessarily occur.

The sort of irregularities to be expected will depend on the periods when the carcinogenic factors are most active and on which of the stages in the process of carcinogenesis are affected. For suppose that the probability, per unit time, of one particular change varies throughout the course of a lifetime, and that the probabilities of the other changes remain constant. In these circumstances, the incidence of cancer at any age will not, as might at first be thought, be proportional to the simple average of the varying probability, but will be proportional to a more complex variable depending on both the above-mentioned factors.

Consider, for example, a varying rate of production of the first of a chain of seven changes. An increased rate of production for a short time during early life will provide a larger number of altered cells to be acted upon by other factors in the future, and will therefore appreciably affect the incidence at, say, age 60. The same increased rate of production of the first change applied for the same short period during middle age will, on the other hand, have little effect on the incidence at age 60, since there will be but little time left—for the altered cells to be acted upon.

Similarly, the incidence at age 60 will be negligibly affected by an increased rate of production of the sixth change in early life. It will, however, be considerably affected by the same change in middle age, when there will be a much larger population of cells which have already experienced five changes and are therefore exposed to the risk of undergoing the sixth.

It is shown in Mathematical Appendix that the formula given above for the incidence at a given age, *t*, is still valid if, for the probability concerned, which was previously assumed to be constant and is now assumed to vary, we substitute a weighted mean of the probability from age 0 to age *t*. The weight depends both on the time of operation of the carcinogenic factor concerned and on the order of the cellular change which it affects. If *t* is the age at which the cancer incidence is observed, *t*_0_ the age at which the subjects are exposed to the factor, and *s* takes a value between 1 and 6 according as the change effected is the first, second, …, or sixth, then the weight is proportional to


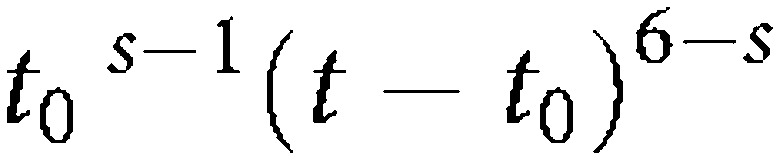


Hence it can be shown that the weight by which a varying probability of the first mutation would be multiplied is highest at age 0 and decreases rapidly throughout life. The weight for the probability of the second change reaches its maximum one-fifth of the way; through the exposure period; that for the third change, two-fifths of the way; and so on. For factors effecting the seventh change, the cancer incidence will be directly proportional to the rate of production of this change at the time of observation, and the previous behaviour of this rate will be irrelevant (i.e., will have zero weight).

Since the weighted mean of the varying probability will in general depend on the age, *t*, the overall incidence at age *t* is no longer proportional to the sixth power of *t*. The incidence will clearly rise more rapidly than the sixth power of *t* whenever the weighted mean is increasing, and more slowly whenever the weighted mean is decreasing. The departures from the sixth-power law already noted for certain sites could be explained on this basis. The deficit in the mortality from cancer of the breast, ovary and cervix uteri in the older age groups (shown in Fig. 4) could, for instance, be attributed to a reduction, during middle age, in the rate of production of one of the late changes. (It may be noted that a similar reduction, during middle age, in the rate of production of an early change would have relatively little effect on the cancer incidence at ages within the span of human life, since the weighted mean would hardly be affected.). The extreme case in which mortality becomes constant after a certain period, as, for example, in cancer of the cervix after the age of 60, can be accounted for by a complete cessation of exposure to factors producing the sixth change. The increased gradient in the curve for cancer of the prostate (Fig. 3) can be attributed to an increase, during middle age, in the rate of production of one of the changes. Such an increase for a late change would be reflected in the mortality rates rising more sharply than would a similar increase for an early change; it would be difficult, on the present data, to distinguish between the effects of a sharp increase in the rate of production of an early change and a smaller increase in that of a late change.

The sites discussed above are those in which the production of cancer is believed to be influenced by endocrine secretions, which vary throughout the course of a lifetime. Lung cancer, on the other hand, is believed to be partly dependent on smoking habits which have changed from year to year. The deficit in mortality below that expected by the sixth-power law, which is shown in Fig. 3 to occur at old ages, can be attributed to the fact that the populations concerned belong to generations which smoked fewer cigarettes than subsequent ones. If mortality at different ages is examined for cohorts born in different five-year periods, the effect disappears.

The concept of carcinogenesis as a multi-stage process also makes it easy to understand the mechanism of the latent period which occurs after exposure to a carcinogenic agent before the appearance of a tumour. For example, Kennaway (1947) has demonstrated that circumcision deferred until the fourteenth year of life fails to give the complete protection against cancer of the penis that it provides when carried out on the eighth day; in other words some change must take place within the first 14 years of life which eventually leads to the development of the disease after a latent period which may be as long as 70 years. From the data of Shrek and Lenowitz (1947), shown in Table 2

**Table 2:** Age at circumcision in patients with and without carcinoma of the penis (after Shrek and Lenowitz, 1947.)

	**No. of men circumcised at ages:**			
**Group**	**0–5 yrs.**	**6–16 yrs.**	**17–35 yrs.**	**No. of men not circumcised**
White men with carcinoma of penis	0	2	5	93
White ‘controls’^a^	18	4	7	125
Ratio between cancer patients and ‘controls’	0.00	0.50	0.71	0.74
Estimated risks relative to risk among uncircumcised	0.00	0.67	0.96	1.00

a The most appropriate control group of the groups cited by Lenowitz and Graham (1946) has been taken to be that of white men with other tumours, excluding ‘veterans’ of World War II.

Coloured men have been excluded from both groups, as the proportions in the two groups were dissimilar and it is likely that the incidence rates among white and coloured Americans are unequal.

, the relative risks of developing penile cancer when circumcision is carried out at ages under 6 years, from 6 to 16 years, from 17 to 35 years, and not at all, are estimated to be respectively 0.00, 0.67, 0.96 and 1.00. On the hypothesis that penile cancer is the end-result of a series of seven successive cellular changes the first of which occurs only in the presence of the prepuce, it can be shown that at age 53 (the average age (calculated from data given by Lenowitz and Graham (1946) and Shrek and Lenowitz (1947)) of all the patients referred to in Table 2) the relative risks of developing the disease when circumcision is carried out at the mid-points of Shrek and Lenowitz's (1947) age periods are 0.30, 0.77, 0.98 and 1.00 (see Mathematical Appendix). Children circumcised during the first six years of life are, however, most likely to have been operated on within the first few weeks, in which case the theoretical risk for this group would be of the order of 0.01. The agreement between the observed and the calculated risks is close and, in view of the smallness of the numbers in the clinical series, must be largely coincidental. Nevertheless, it is fair to say that in this instance the data are consistent with the hypothesis. Another example may be the observation that early age at marriage has a predominating effect in the production of cancer of the cervix uteri (Lombard and Potter, 1950).

A third, the observation of Case (personal communication) that in a group of patients with cancer of the bladder due to industrial hazards, the mean induction time of the tumours—measured from the start of the men's exposure to the risk—was independent of the duration of the exposure. This becomes comprehensible if the industrial carcinogen is presumed to effect the first of a series of changes; the effect of the first few years, exposure would then be predominant and induction time and duration of exposure would appear to be largely independent.

By this discussion it is not intended to suggest that our present findings prove that carcinogenesis is a seven-stage process. Firstly, the sixth-power relationship would result from a multi-stage process with less than seven stages, provided that the rate of occurrence of at least one of the changes is not constant, but increases in proportion to some power of the age. Secondly the relationship holds only over the limited age range of about 25 to 74 years. Whether it would be found to hold at higher ages if mortality data were more accurate is a matter for conjecture. On the other hand, if the present data were held to be equally accurate at all ages (including the oldest) it would be possible to obtain a quite different mathematical relationship. Thirdly, mechanisms other than that of a multi-stage process can also be postulated to account for the observed relationship—and one has, in fact, been suggested by Fisher and Hollomon (1951). Moreover, some other diseases show similar types of increase with age (e.g., cerebral haemorrhage, coronary thrombosis and gastric ulcer) and it is difficult to believe that they should all be dependent on the same type of mechanism.

Berenblum and Shubik (1949), in summarizing the results of their experiments, have, however, stated that “whatever interpretation is adopted as a base line for research, the recognition that carcinogenesis is at least a two-stage process, should invariably be borne in mind”. It is, therefore, natural to see whether a two-stage process—or even a more complex multi-stage one—can account for the human data. From the analysis that has been made here, it would seem that a complex process of perhaps six or seven stages could account for:

1. the rapid increase in mortality with age observed in cancer of some sites, and
2. the irregularity in the increase in cancer of some other sites;
3. the long latent period observed after exposure to a carcinogen before a tumour develops;
4. clinical observations such as the failure of circumcision carried out in adolescence to protect against cancer of the penis, and
5. the experimental finding that cancer incidence tends to be proportional to the concentration of the applied carcinogen.

## SUMMARY

The theory that human cancer is the end-result of several successive cellular changes is tested by examining the age specific mortality rates for 17 types of cancer. On the supposition that the carcinogenic factors responsible have remained approximately constant over the past 75 years, the rates for cancer of the oesophagus, stomach, colon, rectum and pancreas in men and for cancer of the stomach, colon, rectum and pancreas in women accord, in general, with the theory.

The mortality rates for cancer of the lung, bladder and prostate in men and for cancer of the lung, breast, ovary and cervix and corpus uteri in women also accord with the theory, if it is postulated that the carcinogenic factors responsible have varied in strength.

A formula has been obtained which can be used to weight the strengths of the carcinogenic factors at different periods and it is shown that the time when the strength of the factors responsible for the individual changes is of greatest importance varies according to which change in the series is affected. The conclusion provides a possible explanation for the observation that circumcision exerts an important protective effect against the development of cancer of the penis only if it be carried out early in life.

## Mathematical Appendix

Suppose that, when a cell (or its lineal descendants) has experienced exactly *r* − 1 changes, the probability of occurrence of the *r*th change, in that line of descent, is *p*_*r*_ per unit time. Then the probability that the *r*th change occurs in the short time interval (*t*, *t*+*dt*) is, asymptotically,

as *t* → 0. This result will be valid for large values of *t* (of the order of a human lifetime) provided that *p*_1_*t*, *p*_2_*t*, …, *p*_*r*_*t* are all sufficiently small (as could be assumed in an application of this theory to human cancer).

The incidence rate per person is obtained by multiplying (1) by *N*, the mean number of lines of descent per person.

A rigorous proof of (1) is given by Armitage (1953); a simpler proof is as follows. If no restriction is placed on the order in which the changes are to occur, the probability that the first *r* − 1 changes occur sometime in the interval (0, *t*) will be

There are (*r* − 1)! possible orders in which these *r* − 1 changes could occur. Since each *p* is assumed to be very small, any change, if it occurs at all, will almost certainly occur once only. Furthermore, any change is equally likely to occur at any instant in the interval (0, *t*). Consequently, all the possible orders are equally likely, and any one order has a probability

In particular, this is the probability of the order in which we are particularly interested. Given that *r* − 1 changes have already occurred, the probability that the *r*th occurs in the small interval (*t*, *t*+*dt*) is *p*_*r*_ *dt*. Hence the total probability that the *r*th change occurs in (*t*, *t*+*dt*) is

In the preceding argument we have assumed that all the *p*'s are constant. Suppose now that one of these values, say *p*_*s*_, varies with time. More precisely, we assume that the probability that the *s*th change occurs in the small interval (*t*_0_, *t*_0_+*dt*_0_), given that *s*−1 changes have already occurred, is *p*_*s*_(*t*_0_) *dt*_0_ where *p*_*s*_(*t*_0_) is a function of *t*_0_. All the other *p*'s are assumed to be constant. Then the probability that the *s*th change occurs in the interval (*t*_0_, *t*_0_+*dt*_0_), and the *r*th in the interval (*t*, *t*+*dt*), is the product of

the probability that exactly *s*−1 changes occur in the interval (0, *t*_0_); this is *p*_1_…*p*_s−1_*t*_0_ ^*s*−1^/(*s*−1)!;
the probability that, given (i), the *s*th change occurs in (*t*_0_, *t*_0_+*dt*_0_); this is *p*_*s*_(*t*_0_) *dt*_0_; and
the probability that, given (ii), the *r*th change occurs in (*t*, *t*+*dt*); this is *p*_*s*+1_*p*_*s*+2_ … *p*_*r*_(*t*−*t*_0_)^*r*−*s*−1^ *dt*/(*r*−*s*−1)!.

Thus, the probability that the *r*th change occurs in (*t*, *t*+*dt*) is

where *p̄*_*s*_(*t*) is the weighted mean of *p*_*s*_(*t*_0_) during the period (0, *t*), the weight at time *t*_0_ being proportional to

On substituting *r*=7, we obtain the expression given earlier in the paper.

It is easy to show that *w*(*t*_0_) is a maximum when *t*_0_=(*s*−1)*t*/(*r*−2). Substituting *r*=7 and *s*=1, the maximum weight occurs when *t*_0_=0; this means that if *p*_1_ changes with time, the incidence rate at any given age is proportional to the weighted mean of *p*_1_ over the whole period of exposure, the weight being greatest near the beginning of the period. Similarly, substituting *r*=7 and *s*=2, 3, 4, 5 and 6, we find that the maximum weight occurs when *t*_0_=*t*/5, 2*t*/5, 3*t*/5, 4*t*/5 and *t*, respectively.

In applying this theory to the data of Lenowitz and Graham (1946) and Shrek and Lenowitz (1947), we must assume that the distribution of the ages at circumcision in the patients without penile cancer is representative of that in the population from which the penile cancer patients have been drawn; on this assumption, the data of Table 2 provide valid estimates of the relative risks of penile cancer in the four groups. We shall ignore the variation in age amongst the patients, and consider the expected incidence of penile cancer at *t*=53 years (the average age of all the patients referred to in Table 2). We consider a theoretical model in which *r*=7 and *s*=1; and in which *p*_1_ is constant for an uncircumcised person, but becomes zero as soon as circumcision is carried out. For each of the four age groups shown in Table 2, we assume, for simplicity, that circumcision takes place at the mid-point of the group, i.e., at 3, 11.5, 26.5 and 44.5 years, respectively. From the theoretical result proved above, the incidence at *t*=53 is proportional to the weighted mean of *p*_1_ over the period of exposure, the weight corresponding to exposure at age *t*_0_ being (53−*t*_0_)^5^. The incidences for the four groups are therefore proportional to

where *T* is the mid-point of each group. Using the values of *T* given above, the incidences are found to be proportional to 0.30, 0.77, 0.98 and 1.00.

A more realistic assumption would be that the men in the first group were almost all circumcised within the first few weeks of life; on this assumption, the theoretical risk for this group would clearly be very small.
